# Peeking into the mysterious world of *Trypanosoma cruzi* and Chagas disease

**DOI:** 10.1590/0074-02760210193chgsa

**Published:** 2022-05-06

**Authors:** Andréa M Macedo, Sérgio DJ Pena

**Affiliations:** 1 Universidade Federal de Minas Gerais

The discovery of Chagas disease (CD) by Carlos Chagas dates to just 112 years ago. However, the finding of *Trypanosoma cruzi* in nine-thousand-year mummies in the Andean coast in Chile, together with phylogeographic studies of the taxon, suggest that the people of Latin America live with this disease since our earliest ancestors first inhabited the continent.^1^ Moreover, it is reasonably accepted that the bat ancestral strains of *T. cruzi* date to just before or soon after the separation of the Gondwana supercontinent, thus adding a few more million years to the history of the parasite.

Zingales and Bartholomeu start their excellent article with a nice brief review of the evolutionary history of *T. cruzi* populations and their encounter with the American peoples.

Although much has been revealed by science in this more than a century of deciphered history, many mysteries still challenge the complete understanding of many aspects of this parasitosis. Especially, we have selected three areas for our comments: (i) the origin of the strains or, as agreed, call them DTUs (discreet typing units); (ii) the occurrence of sex in the taxon, and (iii) the relevance of these previous two factors to the pathogenetic mechanisms involved in the determination of the different clinical presentations of CD.

Detailing each of these mysteries or repeating the points so well covered in the article in question is beyond the scope of this short comment. In this fashion, our strategy was to point briefly some aspects complementary to those already so well reviewed by the authors.

*The strains or DTUs* - Certainly, an aspect that still needs to come to a definition is whether DTUs are true phylogenetic lineages. Although there is still some controversy in the literature, the consensus appears to be that it is. Of the seven DTUs currently recognized by the scientific community, six infect humans with greater or lesser success and are relevant to the determination of the symptomatic forms of the disease (TcI-TcVI). The last one (TcBat) is basically found in bats, except for a single report in a 5-year-old child.^2^

Unraveling the phylogenetic relationships between strains and establishing which ones would be in fact the ancestral lineages are all important aspects still to be settled. If on the one hand it is reasonably accepted that the *T. cruzi* taxon may have originated from a bat trypanosome from the African continent which has diversified and widespread geographically across Central and South America (bat-seeding hypothesis), on the other hand it is most likely that this ancestral bat trypanosome is quite distinct from the current TcBat, since this DTU seems to have originated much more recently in the history of *T. cruzi*.^3^

In fact, when *T. cruzi marinkellei* is used as an outgroup in the construction of phylogenetic trees of the taxon, most of the data indicates that TcII was the DTU that first diverged from the ancestral lineage, followed by the current TcIV, TcIII, then TcI and TcBat, the latter two almost simultaneously, seemingly constituting sister lineages.^4^

*The sex* - Another debate yet to come to a conclusion is the occurrence, or not, of sex in *T. cruzi*. There appears to be no longer discussion whether there are hybridisation events, genetic exchange or homologous recombination ― these all seem to be well accepted by most. Even the occurrence of meiosis has been confirmed in the taxon.^5^ What remains to be established is the frequency and significance of these events for the evolution of the parasite and for CD. In fact, the latest trends indicate the occurrence of sex at a much higher frequency than initially expected, perhaps comparable to truly sexual populations.^5^ ^,^ ^6^

The long-lasting theory of clonal evolution for the taxon actually resulted from the fact that most genetic exchanges in *T. cruzi* occur between individuals from the same population and from the same lineage, who are genetically more similar to each other. Hybridisation events between individuals of different strains seem to be rare. Among these, however, we highlight the successful evolution of hybridisation events (at least two) between parasite lineages TcII and TcIII, which originated the current hybrid lines TcV and TcVI, very well adapted to human infections.^6^

Additionally, hybridisation events between TcIII and TcIV individuals cannot be discarded and may explain the detection of mitochondrial introgression between these lineages and the occurrence of parasites, identified as TcIV, but much more similar to TcIII than other parasites of the same DTU.^4^

*The pathogenic mechanisms* - But without doubt, the greatest mystery yet to be unraveled involves the pathogenetic mechanisms associated with the different clinical forms of CD. Indeed, the factors that determine why some infected individuals develop clinical forms of the disease, while others remain asymptomatic for life are not completely known. Differences between strains are certainly important, since CD is more severe in regions of predominance of TcII and TcVI, and the distribution of digestive forms seems to be especially associated with the circulation of TcII and TcV lineages. But these associations alone do not explain the differences observed in the clinical manifestations of individuals.

It has been widely shown that the distribution differences of *T. cruzi* clones in organs of patients and infected animals is closely related to the tissue injury and thus, putatively, to the clinical form of the disease. This is the basis of the Clonal Histotropic Model, which was proposed by us in the late 1990s and which persists today.^7^ ^,^ ^8^ ^,^ ^9^

Recent results of gene expression in infected animals with mixtures of populations of *T. cruzi* show that the mixture of clones infecting an organism is capable of inducing different tissue-specific responses, such as reduction of the oxidative metabolism and production of reactive oxygen species (ROS) versus the increased expression of genes associated with Th1 response of the host. This results in the success or failure of maintenance of infection of different clones in the tissues of the host. This phenomenon, often referred to in the literature as “differential tissue tropism”, explains the different clinical forms of the disease as a result of the interaction of the parasite and host genomes, and the ability of specific *T. cruzi* clones to induce genes involved in signaling, proliferation and evasion of the immune system, thus determining the survival of some populations of parasites over others and their permanence in the target tissues of the host, causing tissue damage and, consequently, establishing the clinical form of the disease.^10^

Comments on the article: Zingales B, Bartholomeu DC. *Trypanosoma cruzi* genetic diversity: impact on transmission cycles and Chagas disease. Mem Inst Oswaldo Cruz. 2021; e210193.
